# Micro- and nanoplastics in gastric cancer: A narrative review

**DOI:** 10.1016/j.toxrep.2026.102358

**Published:** 2026-10-16

**Authors:** John Singh Russel, Sulekha Khute, Jinwon Choi, Sohyun Park, Muntajin Rahman, Amama Rani, Bonglee Kim, Paranthaman Subash

**Affiliations:** a School of Pharmacy, Joy University, Vadakkankulam, Tirunelveli Dist, Tamil Nadu, 627 116, India; b Department of Pharmacy, Sri Shanmugha College of Pharmacy, Sankari, Salem, Tamil Nadu, 637 304, India; c Department of Pathology, College of Korean Medicine, Kyung Hee University, Seoul, 02447, Republic of Korea; d Korean Medicine-Based Drug Repositioning Cancer Research Center, College of Korean Medicine, Kyung Hee University, Seoul, 02447, Republic of Korea

**Keywords:** Microplastics, Nanoplastics, Stomach neoplasms, *Helicobacter pylori*, Oxidative stress, Signal transduction, Environmental pollutants, Carcinogenesis

## Abstract

Micro- and nanoplastics (MNPs), defined as plastic particles smaller than 5 mm and 1 μm, respectively, are ubiquitous environmental contaminants detected in human tissues and typically ingested through food and drinking water. No threshold for adverse health effects has been established. This narrative review synthesizes current evidence on the molecular mechanisms, interactions with *Helicobacter pylori*, and experimental findings from 2D and 3D gastric cancer models relevant to MNP-induced gastric carcinogenesis. Particle size, shape, polymer type, surface charge, and weathering status influence toxicity. Proposed mechanisms include oxidative stress, mitochondrial dysfunction, chronic inflammation, genotoxicity, gastric dysbiosis, and ferroptosis, mediated by dysregulation of NF-κB, Wnt/β-catenin, MAPK, PI3K/Akt/mTOR, and cGAS-STING signaling. Nanoplastics (~100 nm) may induce ferroptosis via Fosl1 and p53, whereas larger microplastic fragments may activate YAP signaling and promote proliferation. MNPs may also carry persistent organic pollutants and toxic metals and may act synergistically with *H*. *pylori* to potentiate mucosal damage. In conclusion, MNPs can plausibly promote gastric carcinogenesis through mechanisms demonstrated in experimental systems, but a causal role in human gastric cancer has not been established. Standardized analytical methods, environmentally realistic exposure models, and large-scale epidemiological studies are needed.

## Highlights

• Micro- and nanoplastics (MNPs) are emerging health hazards.
• MNPs can induce oxidative stress and gastric inflammation.
• Plastic particles alter key gastric carcinogenesis pathways.
• Interactions with Helicobacter pylori worsen gastric damage.
• Standardized detection and epidemiology studies are needed to assess risk.

## Graphical Abstract

**Figure:**
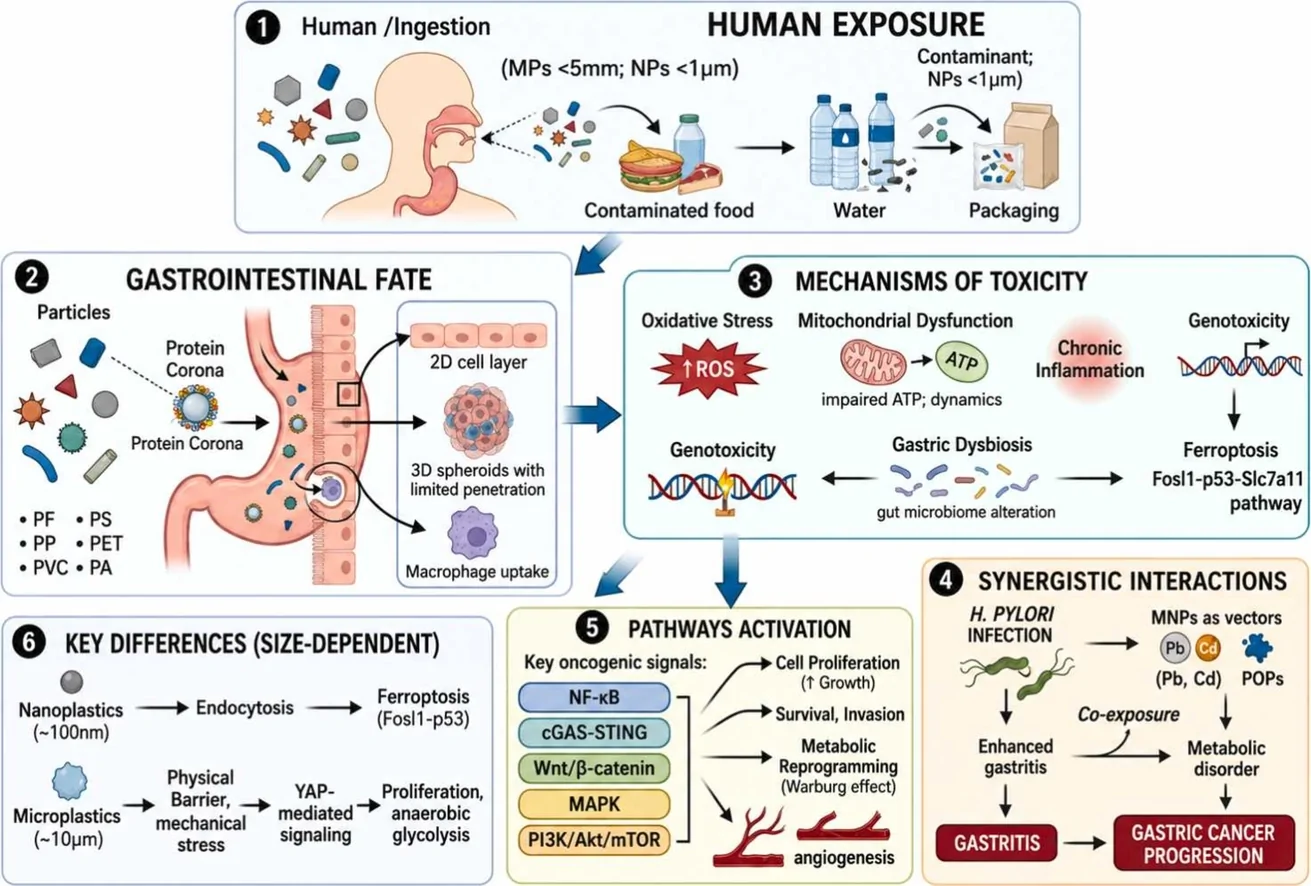

## 1 Introduction

Micro- and nanoplastics (MNPs) are pervasive environmental pollutants generated by the fragmentation of larger plastic products and by the manufacture of plastics at the micro- and nanoscale [1]. Microplastics (MPs; <5 mm) and nanoplastics (NPs; <1 μm) have been detected in marine and freshwater environments, terrestrial ecosystems, air, and a wide range of food products [2]. Human exposure occurs mainly through ingestion of contaminated food and drinking water, with additional routes via inhalation of airborne particles and dermal contact with personal care products. Estimated daily intakes range from thousands to more than 100,000 particles, depending on diet and local environmental contamination [3]. MNPs have been detected in human blood, feces, placenta, and lung tissue, indicating systemic distribution [4]. As emerging risk factors, MNPs are suspected of contributing to inflammation, oxidative stress, and carcinogenesis, including gastric cancer [5]. However, their detection and quantification remain challenging because of their minute size, morphological diversity, and complex polymer composition, such as polyethylene (PE), polypropylene (PP), and polystyrene (PS).

Gastric cancer (GC) is the fifth most common cancer in the world and one of the major cancer-related death causes accounting for approximately 14% of cancer deaths in the world. However, the incidence rates have been different by region and population in the decade compared to the previous decade [6]. Gastric cancer is divided into two categories—cardia and noncardia gastric cancers—which have different precursor lesions and etiological factors. *H pylori* infection is considered the most important risk factor for noncardia GC, while cardia GC is believed to be related to a Western lifestyle, such as obesity and gastroesophageal reflux disease (GERD) [7]. The main route of MNP entry into the human body is through contaminated food and water. These particles migrate across the gastrointestinal mucosal barrier and into the blood stream where they may be carried to tissue and organs [7], [8]. Recent research has also shown that MNPs appear in human blood, faeces, placenta and lung tissue, suggesting that there is extensive systemic exposure [9]. Although MNPs are increasingly being identified in human tissue, their health effects especially on gastric carcinogenesis remain poorly understood [10].

### 1.1 MNPs as emerging risk factors

Five mechanistic hypotheses, which are not mutually exclusive, have been proposed to explain how MNPs may contribute to gastric carcinogenesis. Each is summarized below.

(i) Cytotoxicity due to direct damage of cells. The gastric epithelial cells can endocytose MNPs, especially nanoscale, producing excessive amounts of reactive oxygen species (ROS), which surpass the epithelial cells antioxidant capacity. This oxidative burden leads to lipid peroxidation, protein oxidation, and DNA strand breaks, as well as mitochondrial dysfunction which contributes to further increase of ROS production and to energy metabolism dysfunction of cells [11].
(ii) Chronic inflammation. Exposure to MNPs induces a pro-inflammatory immune response by triggering innate immune signaling and the NF-κB and NLRP3 inflammasome pathways that lead to the release of pro-inflammatory cytokines like IL-6 and IL-1β. This persistent subacute inflammation can form a gastric microenvironment that is pro-tumorigenic over time, similar to that seen in *H. pylori*-associated gastritis [12].
(iii) Gut/ Gastric Microbiome Disruption. Ingestion of MNP has been demonstrated to cause changes in the composition and diversity of the microbial community of the gut and stomach, decreasing the abundance of beneficial commensal species and increasing the abundance of potentially pathogenic species. This dysbiosis may lead to the impairment of mucosal barrier function, raise gut and gastric permeability and allow the translocation of bacterial products and toxins, which further contribute to the local inflammation [13].
(iv) Endocrine disruption. There are often chemical additives in MNPs (adsorbed or contained) that can leach out under physiological conditions, such as plasticizers, stabilizers, and flame retardants. The majority of these additives have been identified as known or suspected endocrine disruptors with the potential to affect hormonal signaling pathways related to proliferation and tissue maintenance of the epithelium, but their specific function in gastric carcinogenesis is not well understood and requires further research [14].
(v) Synergistic interaction with gastric carcinogens already in use. MNPs may serve as a "second hit" that exacerbates the carcinogenic process associated with the main risk factor for noncardia gastric cancer, namely *H. pylori* infection. The combined effect of co-exposure studies indicate that *H. pylori* and MNPs when co-exposure together causes more severe gastric mucosal damage, oxidative stress and release of inflammatory cytokines than when either is present on its own, which may lead to a stage of gastric mucosal dysplasia and malignancy at higher rate [15].

### 1.2 Rationale and novelty of this review

The role of MNPs in gastric carcinogenesis is still emerging and there are several questions unanswered. This narrative review aims to assess the current available evidence and to identify areas for future research on the molecular mechanism(s) whereby MNPs are involved in gastric cancer. Dietary MNP exposure may induce oxidative stress, inflammation, barrier disruption, and other systemic effects, although definitive human health risks remain uncertain [16]. In particular, it investigates how the surface properties, type of polymers and particle size affect the carcinogenic potential of MNPs in the gastric environment [17]. In addition, the role of MNPs in H. pylori infection as possible ‘second hits’ and in vitro relevance to human health is discussed [15]. For the first time, we systematically discuss the molecular pathways, synergistic interactions with H. pylori and the use of 3D gastric cancer models for studying MNP-induced carcinogenesis. Existing evidence is examined and used to assess their strength and identify areas of uncertainty and future research.

The review covers in detail the molecular pathways and interaction of MNPs in the gastric carcinogenesis and the role of 3D gastric cancer models in understanding the synergistic role of MNPs with *H. pylori*. Common in water, food and consumer products, they are ingested by humans and enter through their gastrointestinal system, but the effects on their gastrointestinal system have not been well studied. Current knowledge about the biological effects of MNPs and their possible modifying effect as second hits in gastric cancer development, in addition to the first hit from *H. pylori* infection, is assessed. Several mechanisms were mentioned such as oxidative stress, mitochondrial damage, ferroptosis, activation of oncogenic pathways (NF-κB, cGAS-STING, Wnt/β-catenin, MAPK, PI3K/Akt/mTOR) [18], [19]. We also look at the differences in toxicity with respect to size of MNP, polymer type, and results from the 2D and 3D cancer models; and ultimately consider the synergistic effects of MNP, *H. pylori*, and other environmental pollutants.

## 2 Methods

MNPs as a potential risk factor in human gastric carcinogenesis, with a focus on the molecular mechanisms of toxicity, interactions with *H. pylori*, and findings from 2D/3D experimental gastric cancer models. Because the aim was a mechanistic synthesis rather than a quantitative pooled estimate, a formal systematic review protocol was not applied. Relevant literature was identified by searching PubMed, Scopus, and Web of Science for articles published up to 2026. The search combined the terms "microplastics," "nanoplastics," "gastric cancer," "stomach neoplasms," "*Helicobacter pylori*," and "mechanisms" using Boolean operators like Scopus, and Web of Science for articles published up to 2026, using keywords such as 'microplastics,' 'nanoplastics,' 'gastric cancer,' and 'mechanisms'. Searches were restricted to articles published in English and to original research articles and relevant review articles. Reference lists of key articles were manually screened to identify additional studies that met the eligibility criteria. The Scopus database search identified 256 records, of which 248 were published in English. The eight non-English records were excluded based on the predefined language criterion. The English-language records were subsequently screened for relevance to the toxicological effects of MNPs on gastric tissue, with particular emphasis on molecular mechanisms and human health relevance. Studies were excluded if they focused exclusively on non-gastric endpoints, lacked relevant mechanistic or quantitative outcomes, or were not available in full text. For each eligible study, a standardized data extraction form was used to record: (i) classification of MPs/NPs; (ii) particle properties, including polymer type, size, shape, surface charge, concentration, and aging status; (iii) experimental model, including in vitro cell lines, in vivo animal models, and three-dimensional culture systems; (iv) source and exposure conditions; and (v) molecular endpoints and mechanisms of toxicity, including oxidative stress, inflammatory cytokine responses, signaling pathway activation, DNA damage, and cell death. Data extraction was performed by two reviewers, and the extracted information was checked for consistency and completeness before inclusion in the final synthesis. The strength of evidence was assessed based on study design, sample size, methodological quality, and consistency of findings across studies.

### 2.1 Classification MPs/NPs

MNPs are generally categorized by size, shape, plastic type, and source (primary or secondary). In this study, we mainly distinguished between primary and secondary MNPs: primary MNPs are intentionally produced at the micro- or nanoscale, whereas secondary MNPs are derived from the degradation of macroplastic materials via physical, chemical, and biological routes. The most prevalent polymers in environmental samples are polyethylene (PE), polypropylene (PP), polystyrene (PS), polyvinyl chloride (PVC), polyethylene terephthalate (PET), and polyamide (PA). In the environment, these anthropogenic polymers exhibit diverse behaviors depending on several characteristics, including density, crystallinity, hydrophobicity, and degradation rates. Furthermore, their environmental and organismal toxicities differ [20].

The nomenclature used to define the plurality of MNPs is often confusing. A key dimension in classifying MNPs forms is identifying whether they are NPs or MPs. Another important aspect of discriminating between different forms of MNPs is whether they are fragments, fibers, spheres, films, or irregularly shaped particles. Moreover, virgin or aged/toxic weathered particles with different surface properties have been found to be distinct [21], [22]. Fig. 1 summarizes the six most common plastics, showing their resin identification codes, monomer composition, (semi)crystalline morphology, typical uses, and average density (g/cm³) for PET, high-density polyethylene, PVC, low-density polyethylene, PP, and PS.

**Fig. 1:**
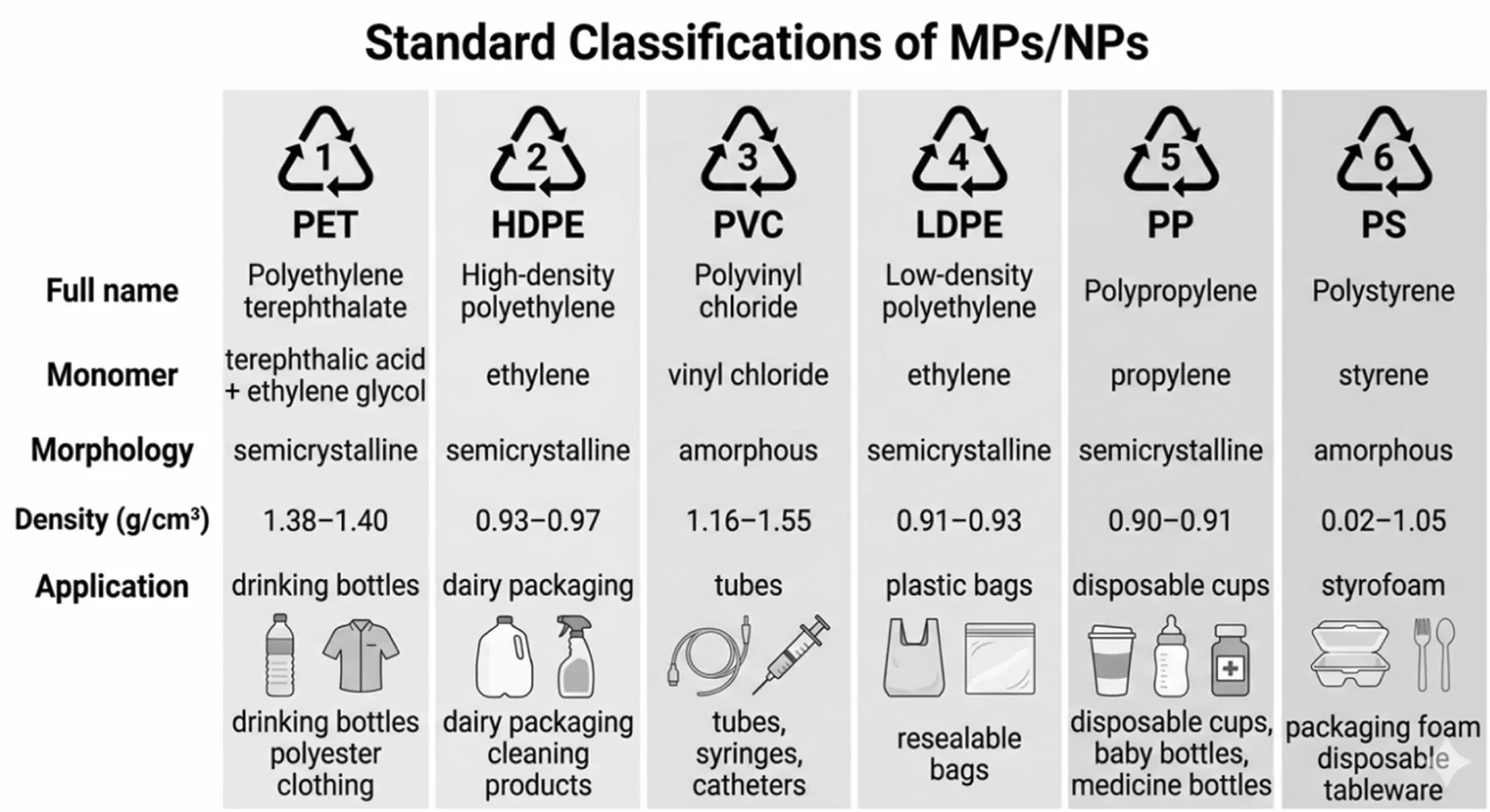
Classification scheme for MPs/NPs based on resin codes, polymer types, monomer compositions, densities, and applications.

Based on their dimensions, MNPs can be distinguished as NPs (less than 1 μm) and MPs (1 μm–5 mm). MNPs are also categorized by their diverse geometric shapes, including fragments, fibers, nanospheres, films, and irregular particles. Virgin and weathered (aged) particles can be distinguished based on their surface properties. The toxicological impacts, cellular internalization, bioavailability, and potential carcinogenic effects of MNPs are primarily governed by these specific physicochemical properties.

### 2.2 Properties of MNP

The properties of MNPs, such as size, shape, polymer (coating) type, surface charge, crystallinity, and weathering degree, play important roles in their environmental behavior, uptake by organisms, and toxicity. MNPs rapidly adsorb proteins and biomolecules from biological fluids, forming a protein corona that affects cellular uptake, immune responses, biodistribution, and toxicity. The composition of the protein corona varies with environmental conditions and influences the biological interactions of MNPs with cells. The physicochemical properties and toxicities of different MNPs are summarized in Table 1, and their interactions with organisms and the environment are discussed based on the available literature.

**Table 1:** Physicochemical characteristics and toxicity of MNPs.

**Particle Size**	**Shape**	**Surface Charge**	**Polymer Type**	**Toxicological Effect**	**Ref**
≤ 1 µm (NPs)	Irregular/spherical	Positive	PE, PP	High cellular uptake, oxidative stress, genotoxicity	[23]
1–5 µm (MPs)	Fibrous/fragmented	Negative	PS, PVC	Barrier disruption, inflammation, cytotoxicity	[24]
> 5 µm	Irregular/aggregated	Neutral	Mixed polymers	Lower uptake; mechanical irritation, immune activation	[25]
Variable	Aggregated clusters	Mixed	Biodegradable vs. synthetic	Biodegradable: less toxic; Synthetic: persistent, more toxic	[26]

In addition to the inherent physicochemical characteristics, properties related to digestion and intestinal barrier passage should be considered. NPs exhibited greater potential to inhibit digestion-related enzymes than MPs. They can also penetrate intestinal barriers more than their larger counterparts and possibly interact with the gut microbiota. Consequently, they can have both local and systemic effects through chemical leaching, nutrient interference, and dysbiosis, attributable to MPs small size [27].

The internalization of MNPs into cells is a complex process governed by the specific physicochemical properties of the MNPs (e.g., size, shape, surface charge, and protein corona) and the dynamic membrane of the target cells. MNPs uptake predominantly occurs via endocytic processes, namely phagocytosis, macropinocytosis, and clathrin- or caveolae-mediated endocytosis. In general, NPs are more efficiently taken up than larger MPs [28]. During gastrointestinal digestion, MNPs undergo several transformations, including changes in aggregation, a shift from neutral to negatively charged surfaces, and the formation of a protein corona. Digestion-released MNPs can increase their uptake by intestinal epithelial cells and macrophages. In vitro studies using Caco-2 intestinal epithelial cell monolayers and a macrophage co-culture model suggested that digested MNPs can disrupt intestinal barrier function, induce inflammation, and activate the NF-κB/NOD-like receptor protein 3 inflammasome, resulting in the production of pro-inflammatory cytokines, including IL-6 and IL-1β [29]. Interestingly, the ingestion of MNPs may not only directly interfere with the action of digestive enzymes but also induce shifts in the gut microbiota, thereby contributing to a dysbalance (dysbiosis) which seems to enhance intestinal permeability. These effects may be particularly relevant in gastric and intestinal tissues, where persistently ingested NPs can drive long-lasting inflammation, contributing to carcinogenesis.

### 2.3 Experimental evidence

*In vitro* and *ex vivo* studies have provided evidence of MNPs effects on gastric cancer progression and development. NPs uptake by gastric cancer cells led to mitochondrial damage, activation of the cGAS-STING and NF-κB pathways, increased HIF-1α levels, and promotion of gastric cancer cell survival. MNPs exposure induced inflammation and pyroptosis in normal gastric epithelial cells. Importantly, both PE and PP-MPs enhanced the proliferation of gastric cancer cells in two (2D) and three-dimensional (3D) cultures, with a more pronounced effect observed in 3D cultures [30]. Among the tested MPs, PP-MPs induced the strongest gastric cancer growth-promoting effects. However, the penetration of MNPs into preformed dense tumor spheroids was limited. While experimental studies have indicated a potential MNP-induced gastric cancer risk, large-scale epidemiological studies are warranted to confirm the association between MNPs exposure and gastric cancer risk in humans [31].

Experimental studies using gastric cancer models have demonstrated the potential pro-tumorigenic effects of MNPs. We observed that both PE and PP-MPs enhanced the proliferation of human gastric cancer cells in 2D monolayer cultures and promoted the growth of 3D spheroids, which more closely mimic the tumor ecosystem. The most pronounced effect on spheroid growth was observed with PP-MPs after 7 days of incubation. Notably, MNPs showed limited penetration into the dense cores of established tumor spheroids, suggesting that MNPs may primarily promote the progression of established gastric cancers. Importantly, the effects of MNPs exposure were strongly size-dependent. NPs (~100 nm) were efficiently internalized by epithelial cells via endocytosis and induced ferroptosis, a form of iron-dependent lipid peroxidation, through the Fosl1-p53-Slc7a11 signaling axis. In contrast, larger microplastics (~10 μm) create a physical barrier to cell membranes, induce the YAP oncogene signaling pathway, and promote a switch to anaerobic glycolysis in exposed cells [32]. In summary, *in vitro* and 3D model studies indicate that MNPs can simultaneously induce damage to normal gastric epithelial cells and promote the survival and growth of gastric cancer cells.

### 2.4 Sources and exposure

MNPs are ubiquitous environmental contaminants and are ingested by humans [33]. As plastic use becomes more widespread and ingrained in everyday life, there is inevitable exposure through its many uses in packaging, storage, and consumer products that are ingested, such as face masks. As plastic waste breaks down, it forms smaller fragments known as MPs and NPs, which have been reported to permeate and contaminate various media, including freshwater and marine environments, terrestrial ecosystems, air, and the human diet [34]. Much remains to be understood regarding the long-term health risks of MNPs exposure. The majority of exposure is considered to occur through ingestion, whereby MNP contaminants are ingested and transported to the GI tract, where they can interfere with gut commensal bacteria or be broken down into smaller toxic chemicals. Evidence also suggests that MNPs can disrupt the intestinal mucosa, leading to the translocation of plastic across the gut lining and into the bloodstream, potentially leading to localized or systemic effects, including plastic-induced inflammation and even neoplasia [35]. Studies conducted in animal models have observed that MNPs can worsen the symptoms of inflammatory bowel disease and metabolic dysfunction–associated steatotic liver disease and can induce certain types of gastrointestinal cancers [36].

Three entry pathways of human exposure to MNPs have been identified, the most likely being the ingestion route. MNPs may be introduced to the human body via contaminated food, such as seafood, fruits, vegetables and breakfast cereals, or via food packaging materials or drinking water. Another important route is via inhalation, especially via airborne particles emitted from synthetic textiles, tyre wear, and indoor environment. Dermal exposure also can happen when people come into contact with personal care products or cosmetics that include microbeads or other plastic particles [37]. The presence of MNPs in the environment and the potential effects on humans are of increasing concern. They may be primary sources, such as cosmetic products, or secondary sources, which are formed from larger plastic items breaking down, and enter the human body by ingestion, inhalation or dermal contact [38]. MNPs have been found in bottled water, seafood, or the air which can increase the daily intake of particles from many thousands to more than 100,000 particles per day. They have been found in the blood, faeces, placenta and lungs, indicating their systemic distribution and warranting further investigations of their long-term effects on health [39]. MNPs, contaminate the human food chain through various pathways, including soil-to-plant transfer, atmospheric deposition, and contamination during food processing and packaging. Ingested matter is present in many edible plant-based food materials, including fruits, vegetables, and cereal grains. Plants take up these materials mainly through root cracks and via endocytosis. Accordingly, plant-based foods are significant dietary sources for humans [40], [41].

### 2.5 Mechanisms of toxicity

The toxicity of MNPs mainly involves multiple coupled toxic effects, including oxidative stress, mitochondrial dysfunction, persistent inflammation, genotoxicity, epigenetic toxicity, and ferroptosis. Chronic gastritis and gastric carcinogenesis caused by long-term ingestion of MNPs mainly occur in the gastric mucosa of mice. Table 2 shows the associated toxicities of MNPs. The toxicity of MNPs is strongly dependent on size. Nanoplastics were readily internalized by intestinal epithelial cells via endocytosis and could induce ferroptosis by activating the Fosl1-p53 pathway, leading to iron overload, glutathione depletion, inactivation of Glutathione Peroxidase 4, and uncontrolled lipid peroxidation. In contrast, larger microplastics primarily act by physically disrupting intestinal barriers and inducing metabolic changes mediated by the Yes-associated Protein (YAP) pathway [42]. Excessive oxidative stress, energy imbalance, and downstream-associated inflammatory and cancerous signaling pathways may result from MNP-induced mitochondrial dysfunction. It can reduce mitochondrial respiratory function, inhibit mitochondrial dynamics, and repress ATP production, thereby increasing ROS levels [43].

**Table 2:** Oncogenic pathways: molecular effects and associated risks.

**Pathway**	**Molecular Effect**	**Associated Disease Risk**	**Ref**
NF-κB Activation	IκB degradation → NF-κB translocation → cytokine release	Inflammation, autoimmune diseases, cancer	[44]
Mitochondrial Dysfunction	Impaired respiration, Increase glycolysis, altered dynamics	Metabolic, cardiovascular, neurotoxicity	[45]
Epigenetic Changes	DNA methylation, histone modification, gene alteration	Gastric & colorectal cancer	[46]
Genotoxicity	DNA damage, chromosomal changes, apoptosis	Mutations, tumor initiation	[47]
Oxidative Stress	ROS generation, membrane damage	Tissue injury, cancer, aging	[48]

**Critical evaluation:** The evidence indicating oxidative stress as a primary mechanism is strong, however, there are some studies that have suggested conflicting dose-response relationships. Based on some evidence, low concentrations (< 100 μg/mL) may be adaptive and stimulate cellular responses, and high concentrations (> 200 μg/mL) are always toxic. The non-linear dose-response relationship makes it difficult to scale up the data from experiments to human exposure scenarios. Further, whether pristine, monodisperse particles employed in most studies are representative of "environmentally weathered" and/or heterogenous MNPs is unclear.

## 3 Results

### 3.1 Role in gastric carcinogenesis

MNPs have been implicated in gastric cancer development through various mechanisms, including cytotoxicity, chronic inflammation, oxidative stress, disruption of energy metabolism, and DNA damage in gastric cells and tissues. Cytotoxic effects of polystyrene nanoplastics of different sizes (80, 500, and 650 nm) on human gastric fibroblasts. Size- and concentration-dependent cytotoxicity of the NPs was also observed. The smallest 80 nm particles exhibited the highest cellular uptake and caused the strongest cytotoxicity. The toxic effects of MNPs on gastric fibroblasts, including mitochondrial dysfunction, ROS production, DNA damage, and opening of the mitochondrial permeability transition pore, lead to damage to gastric tissue components and impairment of gastrointestinal function [49].

MNP-induced toxicity can be size-, charge-, and functional group-dependent. Through oxidative stress and induced genotoxicity, MNPs can trigger apoptosis and ferroptosis/pyroptosis in biological systems, disrupting normal biological functions and forming a pro-tumorigenic microenvironment. Special attention should be paid to the possible risk of gastric carcinoma in humans [50], [51]. Fig. 2. Schematic illustration of carcinogenesis induced by nanomaterial/MNPs exposure.

**Fig. 2:**
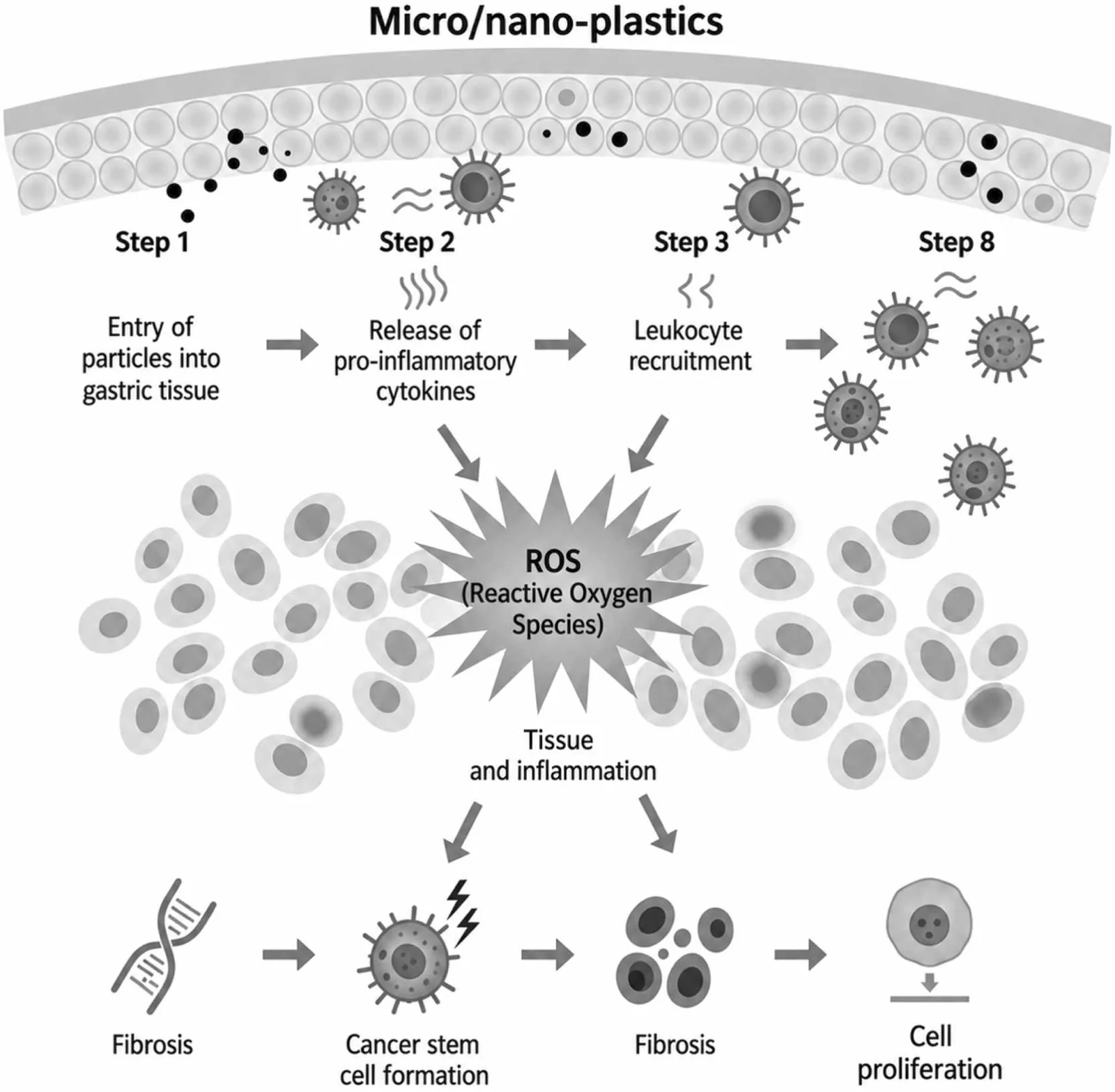
Detailed schematic illustration step-by-step depicting MNPs entry, ROS generation, leukocyte recruitment, cellular damage, and carcinogenesis progression.

### 3.2 Molecular pathways linking MNPs to gastric carcinogenesis

Exposure to MNPs is suggested to be associated with gastric carcinogenesis, mainly by inducing oxidative stress and chronic inflammation, and subsequently modulating various gastric epithelial and stromal cell signaling pathways that promote cancer development. Exposure to MNPs leads to activation of important pathways of cells that are responsible for cancer progression. The Wnt/β-catenin pathway is hyper-activated in gastric cancer which results in increased nuclear β-catenin levels and up-regulation of proliferation, invasion and metastasis-related genes. MNPs increase the pathway by producing reactive oxygen species (ROS). Likewise, ROS activates the NF-κB pathway, which leads to degradation of IκB with subsequent activation of production of pro-inflammatory cytokines. The MAPK pathway (ERK, JNK and p38 kinases) supports cell proliferation and survival, whereas the PI3K/Akt/mTOR pathway, stimulated by oxidative stress, stimulates cell growth and blocks the cell death process. The pathways highlight the involvement of MNP-induced ROS in the field of cancer biology [52], [53], [54].

### 3.3 Interaction with microbiome

Exposure to MNPs can induce DNA damage and mitochondrial dysfunction by generating reactive oxygen species (ROS) and triggering an inflammatory response. Damaged mitochondria can leak cytosolic DNA, activate the cGAS-STING pathway and inducing NF-κB activation, increased hypoxia-inducible factor 1-alpha (HIF-1α), the Warburg effect, angiogenesis, epithelial-mesenchymal transition, and a senescence-associated secretory phenotype, thereby creating a pro-tumorigenic microenvironment and promoting gastric cancer progression in the stomach, with *H. pylori* as the first hit. MNP-induced changes function as second hits to accelerate *H. pylori*-induced gastric carcinogenesis [55], [56].

Exposure to MNPs may increase the risk of gastric carcinogenesis by disrupting the gut microbiota. In the GI tract, MNPs can induce oxidative stress, impair intestinal or gastric mucosal barrier function, and provoke inflammation. Furthermore, gut MNPs exposure can reduce the abundance of certain beneficial microorganisms and increase the abundance of potentially pathogenic species (dysbiosis), thereby enhancing gastric inflammation and reducing gastric mucosal barrier functions. MNPs can also facilitate the transmission of antimicrobial resistance genes and be used as surrogates for microbial interactions in the tumor microenvironment of gastric cancer [57], [58].

### 3.4 Synergistic effects

Exposure to MNPs may also increase the risk of synergistic interactions with other gastric carcinogens. Co-exposure to *H. pylori* significantly increased gastric mucosal damage and inflammation (IL-6), oxidative stress, and lipid metabolism disorders compared to individual exposure. Additionally, MNPs exposure as an environmental “second hit” might promote the persistence of bacterial pathogens, disrupt the gastric mucosal barrier, and induce chronic inflammation, thereby contributing to the progression from gastritis to gastric cancer [15], [59].

In addition to potential toxic effects on living organisms, available data suggest that surface-modified MNPs can interact synergistically with other environmental pollutants. Some studies indicate that MNPs can act as ‘carriers’ for heavy metals (Pb and Cd) and POPs, enhancing their bioavailability, potential for bioaccumulation, and toxicity. Such interactions can also enhance ROS production, gastric mucosal genotoxicity, and stimulate oncogenic signaling pathways (Table 3).

**Table 3:** Synergistic effects of MNPs with co-exposures (Heavy metals, POPs, Cigarette smoke, Arsenic).

**Co-Exposure Agent**	**Interaction with MNPs**	**Combined Effect**	**Ref**
Heavy metals (Pb, Cd)	Carrier effect, increase bioaccumulation	Increase oxidative stress, toxicity	[60]
POPs	Adsorption & transport	Increase bioavailability, oxidative stress	[61]
Cigarette smoke condensate	Synergistic ROS & DNA damage	Genomic instability, carcinogenesis	[62]
Arsenic	Amplified genotoxicity & signaling	Increase cancer risk, mutagenicity	[63]

**Table 4:** Detection techniques for MNPs (FTIR, Raman, SEM, spICP-MS, AFM-IR).

**Method**	**Strengths**	**Limitations**	**Size Range**	**References**
Fourier Transform Infrared	Reliable polymer identification	Low sensitivity for small particles	> 10 µm	[64]
Raman Spectroscopy	High-resolution, non-destructive	Weak signal for nanoplastics, complex preparation	~1–20 µm	[65]
Scanning Electron Microscopy	Excellent surface morphology	Limited chemical information below 1 µm	> 1 µm	[66]
Single Particle Inductively Coupled Plasma Mass Spectrometry	Quantitative analysis in complex matrices	Limited polymer identification	< 1 µm	[67]
AFM-IR	High spatial resolution & chemical mapping	Time-consuming, requires specialized equipment	100 nm – 5 µm	[68]

These synergistic effects underscore the importance of considering combined environmental exposures in gastric cancer risk assessment and prevention.

### 3.5 Identification

To determine exposure and assess potential risks to living organisms, it is essential to identify and quantify MNPs in various environmental matrices and biological samples. Several novel methods have been developed in recent years. Atomic Force Microscopy-Infrared (AFM-IR) Spectroscopy is capable of high-resolution, non-destructive analysis at the submicron spatial scale, allowing polymers, crystallinity, and contamination in particles ranging from 200 nm to several micrometers to be characterized. Therefore, Atomic Force Microscopy-Infrared Spectroscopy can distinguish synthetic MNPs from unwanted biological contaminants, such as diatoms and bacteria, and could be particularly useful for analyzing complex environmental samples [69].

Although there have been rapid advancements in extraction and characterization methodologies for MNPs in aqueous solutions, significant challenges remain in analyzing MNPs in complex organic biomatrices, such as gastric tissues, blood, and feces. There is an urgent need to optimize and validate techniques for the digestion, separation, and concentration of MNPs in these matrices to minimize interference and enable accurate quantification and characterization. Currently, there are no optimized methods for the specific analysis of biological tissues, and methods to accurately quantify trace amounts of NPs in biomatrices remain to be established.

Future work will require novel approaches that combine machine learning algorithms with spectroscopic technologies (such as surface-enhanced spectroscopy) and stable labelling strategies that employ appropriate reference MNPs to accurately detect in complex bio-matrices. From an environmental perspective, green chemistry strategies that exploit olive oil-based emulsions to remove MNPs and other NPs from food, human fluids, and environmental matrices, along with the corresponding sample preparation and purification methodologies, will also be of great importance [70]. Method standardization and large-scale biomonitoring studies are needed to assess MNPs distribution in human tissues, evaluate true exposure risk, and determine possible health effects.

## 4 Discussion

ROS have been shown to regulate different oncogenic signaling pathways such as Wnt/β-catenin, NF-κB, PI3K/Akt/mTOR, and MAPK. In cancer, Wnt/β-catenin pathway is frequently activated, and ROS has been shown to affect β-catenin stabilization and nuclear translocation, leading to increased cell proliferation and survival [71]. Likewise, ROS activation of NF-κB, a transcription factor that plays an essential role in inflammatory response and cell survival, results in the expression of genes which favour inflammation and oncogenesis. ROS also activates the PI3K/Akt/mTOR pathway, which plays a key role in cell growth and metabolism, and promotes survival and growth of cancer cells. Further, activation of the MAPK pathway by ROS results in the expression of genes related to cell growth, differentiation and resistance to cell death. cGAS–STING pathway is a critical part of the innate immune response and is responsible for sensing of cytosolic DNA and triggering downstream signaling events that lead to the production of type I interferon and other cytokines [72]. In cancer, this pathway can both work for and against the cancer. It may help to activate anti-tumor immunity through the improvement of immune surveillance, but certain tumors have developed strategies that utilize cGAS–STING signaling to facilitate their growth and survival. ROS can affect this pathway by inducing damage on DNA which results in the presence of cytosolic DNA and activation of cGAS–STING.

The Warburg effect is a metabolic reprogramming of cancer cells which is marked by an increase in glycolysis, even in the presence of oxygen. The change in metabolism is mostly controlled by hypoxia-inducible factor 1-alpha (HIF-1α), which accumulates in hypoxic conditions or high levels of ROS [73]. By switching the metabolic program of the tumor cell to glycolysis, HIF-1α triggers the upregulation of genes involved in glycolysis, angiogenesis, and cell survival, which facilitates the rapid growth and proliferation of cancer cells and helps them adapt to the tumor microenvironment. MNPs have emerged as a new generation of environmental pollutants that affect human health, particularly through the gastrointestinal tract [74]. After ingestion, MNPs can interact with the gastric microenvironment and cellular signaling networks, which regulates cell proliferation and differentiation. MNPs can modulate several signaling pathways, including the Wnt/β-catenin pathway by generating oxidative stress or modulating the gut microbiota, leading to abnormal cell growth [75]. The NF-κB signaling pathway is involved in inflammation and has been reported to be activated by MNPs, leading to the production of pro-inflammatory cytokines via ROS [76]. The PI3K/Akt/mTOR signaling pathway, which regulates gastric cell homeostasis and modulates nutrient sensing to promote cellular growth and survival, is involved in cell survival and metabolism. This pathway can be modulated by MNPs, which alter cellular stress responses [77]. The MAPK signaling pathway regulates gene expression [78]. These data collectively suggest that MNPs may shift gastric cellular homeostasis to a pro-tumorigenic state.

MNP-induced oxidative stress is activated and transduced into the activation of oncogenic signals through a complex biological process that begins with cells exposure to MNPs in the environment [79]. MNPs can generate ROS through direct interactions with cellular components and the leaching of toxic additives. ROS can cause lipid peroxidation, DNA damage, and protein oxidation by disrupting the balance between antioxidants and free radicals. Elevated ROS levels can activate several signaling pathways, including NF-κB, AP-1, MAPKs, and PI3K/Akt, which participate in cellular homeostasis. However, chronically elevated ROS can activate aberrant oncogenic signals, leading to the transcription of genes involved in cell survival, proliferation, and inflammation [80], [81]. Therefore, persistent redox imbalances may contribute to carcinogenic processes by causing mutations, stimulating cell proliferation, and inhibiting apoptosis [82].

DNA from damaged cells can also activate the innate immune DNA-sensing pathway, cGAS-STING-NF-κB, in the presence of MNPs. Recently, this pathway has been implicated in tumor biology, including GC. The sensor cGAS (cGMP-AMP synthase) detects cytosolic DNA. The second messenger cGMP-AMP, produced by cGAS, activates the transmembrane protein STING. Activated STING recruits and activates two protein kinases, TBK1 (TANK-binding kinase 1) and IKK (IκK kinase), in the NF-κB signaling cascade. Phosphorylated TBK1 and IKK activate the transcription factors nuclear factor kappa B (NF-κB) and interferon regulatory factors (IRFs) [83], [84]. NF-κB activation has been shown to be involved in the development of gastric cancer. For instance, NF-κB can induce the expression of HIF-1α (hypoxia-inducible factor 1α) [85]. HIF-1α is stabilized under hypoxic conditions (low oxygen levels) and activates the transcription of genes involved in angiogenesis (formation of new blood vessels), altered metabolism, and cell survival. Notably, under hypoxic conditions, cancer cells may shift to glycolysis for energy production, even in the presence of oxygen (the Warburg effect), a process promoted by HIF-1α [86], [87].

In addition to the individual effects of MNPs and *H. pylori* exposure, their co-exposure may have synergistic effects on the human body. Co-exposure to MNPs and *H. pylori* can increase gastric mucosal damage and disrupt lipid metabolism. The hostile stomach environment caused by these two agents can increase the inflammatory response [88]. MNPs, owing to their small size and persistence in the environment, can interact with and, depending on their physicochemical characteristics, potentially cross the gastric mucosa and facilitate the transport of associated contaminants, thereby increasing oxidative stress and inflammation caused by *H. pylori*. In addition, both MNPs and *H. pylori* affect lipid metabolism by interfering with fatty acid oxidation and cholesterol homeostasis [89], [90], [91]. However, there is still a limited amount of experimental evidence to support a synergistic effect, and it's not clear whether a synergistic effect will occur at environmentally relevant human exposure levels. However, the bioavailability of associated contaminants associated with an increase in MNPs is dependent on polymer type, aging of particles, surface chemistry, environmental factors, and gastrointestinal physicochemical factors. Thus, the effects of the carriers must not be generalized to all types of MNP or exposure situations.

Furthermore, MNPs can act as vectors to alter the transport, bioavailability, and toxicity of co-occurring pollutants. Many heavy metals and POPs are carried by MNPs as vectors to targeted locations, thereby increasing their toxic effects. For instance, the adsorption of lead and cadmium by magnetite and the subsequent increase in bioavailability or persistence of pollutants in biological systems have been reported [92], [93], [94], [95]. The direct effects of MNPs on cell cultures have been investigated in several experimental models, providing important insights into the cellular responses they elicit. In 3D spheroid models of cancer cells, interactions between MNPs and the tumor cell microenvironment have recently been studied [96]. For instance, MNPs were found to promote the survival of gastric cancer cells, but they failed to penetrate deeply into densely packed 3D tumor cell masses [97], [98]. Although the results seen in 2D monolayers and 3D spheroids can be translated to human gastric tissues, this is not straightforward due to differences in the extracellular matrix, the production of mucus, interactions between immune cells, the architecture of the mucosa, oxygen gradients, and conditions of exposure. Therefore, more physiologically relevant models like organoid, organ-on-chip, and co-culture models, including gastric epithelial cells, immune cells, stromal components, and *H. pylori*, are needed.

As solid tumors grow, MNPs tend to have less effect than earlier carcinogenic processes or at the tumor periphery. The size of MNPs not only affects tumor penetration depth but also the type of cell death that occurs. Nanoparticles that occur (~100 nm) preferentially induce ferroptosis, an iron-dependent process of the accumulation of lipid peroxides in cells, particularly through the transcription factor Fosl1 that sensitizes cells to oxidative stress [99]. On the other hand, larger MNPs (~10 μm) primarily cause mechanical damage to cells, leading to necrosis or apoptosis [100], [101]. These size-dependent effects should, however, be interpreted cautiously because experimental studies frequently use monodisperse, pristine particles at concentrations that may exceed realistic human exposure. Environmental MNPs are generally heterogeneous mixtures that differ in size, shape, polymer composition, surface chemistry, aging status, and associated contaminants.

### 4.1 Methodological limitations and uncertainties

A major limitation in the current MNPs literature is the lack of standardized methods for particle detection, identification, and quantification. Characterization of MNPs in environmental and biological samples is challenging due to their small size, their complex biological matrices, their low concentrations and to the potential contamination that can occur during sample collection and the laboratory processing [102]. The detection limits, polymer identification capabilities and the ability to characterise the number, size and morphology of the particles are different for common analytical techniques such as Fourier-transform infrared spectroscopy (FTIR), Raman spectroscopy, pyrolysis–gas chromatography/mass spectrometry (Py-GC/MS) and thermal analysis [103]. Specifically, the conventional spectroscopic methods can underestimate the number of particles below their effective detection limits, while the mass-based methods can yield the mass of the polymer without information on the number, size distribution of particles, or particle shape.

Because of this, it is often difficult to directly compare reported MNPs concentrations from various studies [104]. The sampling process, digestion, recovery, contamination controls, reporting units, and various analytical platforms can cause significant uncertainty. But the fact that there are no harmonized quality control procedures or reference materials makes it difficult to compare environmental and animal studies with human studies. Future investigations should thus employ validated and standardized analytical workflows, procedural blanks, recovery experiments, positive controls and suitable measures for contamination control, and reporting the particle size distribution, polymer composition, polymer morphology, concentration, and the analytical detection limit [105].

Dose estimation represents another major uncertainty. Numerous experimental studies use very high concentrations of MNPs, typically single polymer, spherical, pristine particles. These types of experimental designs are appropriate for elucidating mechanisms but are not necessarily representative of chronic human exposure to chemically weathered, aged and heterogeneous particles. Environmentally relevant concentrations and exposures, such as repeated low-dose exposures, mixed polymer particles, weathered particles and contamination of food-related particles should be the focus of future toxicological studies [106]. Particular emphasis should be placed on using complementary metrics such as particle number, particle mass and particle surface area, and where applicable, polymer-specific concentration, when reporting doses, to enable comparison of doses across studies.

A major uncertainty is the dose-response relationship between exposure and the biologically effective dose in gastric tissues. Human absorption of MNPs after ingestion has not been fully characterized and the percent of MNPs retained in the stomach, transported throughout the gastrointestinal tract, absorbed across the intestinal epithelium or excreted in the feces is still not well understood. The behavior of the MNPs and cell uptake can be significantly affected by factors like gastric pH, mucus, digestive enzymes, food composition, intestinal transit, particle aggregation and protein corona formation [107]. The study of human biomonitoring, especially longitudinal studies, where measured exposure is correlated with internal tissue concentration and biological outcome, is therefore very important.

Another limitation in the interpretation of MNPs-associated carcinogenicity is the predominance of in vitro and animal evidence. Such association of MNPs exposure with oxidative stress, inflammation, DNA damage or with changed signaling does not imply that exposure to MNPs results in gastric cancer [108]. Long-term studies are needed to see if repeated exposure results in long-lasting inflammation, precancerous lesions, dysplasia, or malignancy in the stomach, especially when other risk factors such as *H. pylori* infection are present. While current evidence highlights potential mechanisms linking MNPs to gastric carcinogenesis, limitations such as heterogeneity of particles and lack of long-term human data remain. Future research should prioritize standardized detection techniques, realistic exposure assessments, and advanced models like gastric organoids. Policymakers should consider regulations on plastic pollution and public health interventions to mitigate risks.

### 4.2 Strengths and limitations

This review integrates molecular pathways, evidence from 3D spheroid models, and the interaction between MNPs and *H. pylori* within a single framework, giving a more comprehensive view of potential effects at the cellular and organismal levels. It also separates mechanistic findings from evidence of human relevance and grades the strength of evidence. Limitations: as a narrative rather than systematic review, study selection was not exhaustively protocol-driven, which introduces a risk of selection bias. The diversity of methods across studies makes results difficult to interpret and compare. Most studies use single-polymer, pristine particles at concentrations that may exceed realistic human exposure, which limits applicability. Human data remain sparse: there are few long-term epidemiological studies and no validated methods for quantifying MNPs in gastric tissue.

### 4.3 Future research priorities and regulatory perspectives

The following are five areas that should be studied in the future. First, there is the need for harmonization of methods for sampling, extraction, identification and quantification of MNPs across countries to provide comparable exposure information [109]. Second, the use of environmentally relevant concentrations and chronic exposure paradigms should be emphasized over reliance on mainly acute high-dose studies in dose–response studies [110]. Third, the inclusion of physiologically relevant gastric models such as human gastric organoids, 3D co-culture systems and organ-on-chip platforms should be included in mechanistic studies, paying special attention to MNPs–*H. pylori* interactions [111]. Fourth, studies to generate epidemiological and biomonitoring data should include the question of whether there is a relationship between tissue or gastrointestinal MNPs burden and gastric inflammation, gastric persistence of *H. pylori*, precancerous lesions, or gastric cancer incidence [112]. Future studies should consider realistic co-exposures of aged MNPs, food-associated contaminants, heavy metals, POPs, and pharmaceutical or microbial stressors.

Regulatory frameworks need to take into account particle size, morphology, surface properties, ageing, additive composition and any associated contaminants when assessing the hazards of MNPs; classification should not be based on polymers alone. There is a need for the standardization of analytical protocols, reference materials, exposure monitoring programmes and reporting requirements for the concentrations of MNPs in food, drinking water and consumer products by regulatory agencies. The vulnerable populations and chronic low-dose exposure should also be included in risk assessment in addition to acute toxicity. While the evidence-base is being built, policies to reduce the unnecessary use of plastic, better waste management, control over plastic degradation and release, and limiting contamination of the environment and food-chain from major environmental, food-chain sources can offer complementary preventive measures.

## 5 Conclusion

MNPs are known environmental contaminants with possible relevance to human health and emerging evidence indicates that exposure may affect biological processes that are involved in gastric carcinogenesis. Once ingested, MNPs may interact with the gastric mucosa, the biological effects of which could be modified by particle size, shape, polymer type, surface characteristics, and chemical impurities. Experimental evidence to date supports MNPs can cause oxidative stress, mitochondrial dysfunction, inflammatory response, gut–gastric microbiome alteration and cellular damage. The effects could be mediated by the activation of existing pathways such as NF-κB, MAPK, PI3K/Akt/mTOR, Wnt/β-catenin, and cGAS–STING. However, a direct causal role of MNPs in human gastric carcinogenesis has not been established.

Some emerging evidence indicates that there might be interactions between the exposure to MNP, *H. pylori* infection and chronic inflammation. These findings are preliminary, however, and must be confirmed in models and in humans that are physiologically relevant. Similarly, genotoxicity, ferroptosis and enhancement of the malignant phenotypes by MNP are still under investigation and should not be considered as being directly causal for the moment. Biological responses may vary according to the type of polymers and particles, but there are not sufficient comparative data to determine if any one polymer has a higher carcinogenic potential.

There are some priorities for future research. First, there is a need for large-scale epidemiological and prospective biomonitoring studies to establish any association between risk of gastric cancer and MNPs exposure in humans. Second, standardized and validated analytical approaches for detection and quantification of MNPs in all human biological matrices should be developed. Third, experimental studies should systematically assess the effects of particle size, shape, type and surface properties of polymer, chemical contaminants and co-exposure to *H. pylori*. Mechanistic results should be confirmed in physiologically relevant human-derived models prior to the conclusion of specific pathways. These strategies will help us understand if exposure to MNP is a risk factor for gastric cancer and if decreasing exposure might be protective.

## Abbreviations

MNPs: Micro- and Nanoplastics (or Microplastics and Nanoplastics)
MPs: Microplastics
NPs: Nanoplastics
GC: Gastric Cancer
*H. pylori*: *Helicobacter pylori*
PE: Polyethylene
PP: Polypropylene
PS: Polystyrene
PVC: Polyvinyl Chloride
PET: Polyethylene Terephthalate
PA: Polyamide
ROS: Reactive Oxygen Species
NF-κB: Nuclear Factor kappa-light-chain-enhancer of activated B cells
cGAS–STING: Cyclic GMP-AMP Synthase–Stimulator of Interferon Genes
Wnt/β-catenin: Wingless-related integration site / Beta-catenin
MAPK: Mitogen-Activated Protein Kinase
MPFs: Microplastic fragments
PI3K/Akt/mTOR: Phosphoinositide 3-Kinase / Protein Kinase B / Mammalian Target of Rapamycin
YAP: Yes-associated Protein
HIF-1α: Hypoxia-Inducible Factor 1-alpha
POPs: Persistent Organic Pollutants
AFM-IR: Atomic Force Microscopy-Infrared
2D: Two-Dimensional
3D: Three-Dimensional

## CRediT authorship contribution statement

**John Singh Russel:** Writing – original draft, Investigation, Data curation, Conceptualization. **Sulekha Khute:** Writing – review & editing, Formal analysis, Data curation. **Jinwon Choi:** Writing – review & editing, Funding acquisition, Formal analysis, Data curation. **Sohyun Park:** Writing – review & editing, Validation, Investigation, Funding acquisition, Data curation. **Muntajin Rahman:** Writing – review & editing, Resources, Funding acquisition, Data curation. **Amama Rani:** Writing – review & editing, Visualization, Funding acquisition, Data curation. **Bonglee Kim:** Writing – review & editing, Project administration, Funding acquisition, Conceptualization. **Paranthaman Subash:** Writing – review & editing, Writing – original draft, Supervision, Methodology, Formal analysis, Conceptualization.

## Ethics approval and consent to participate

None.

## Declaration of Generative AI and AI-assisted technologies in the writing process

During the preparation of this work the author(s) used Grammarly and Paperpal in order to improve language readability. After using this tool, the author(s) reviewed and edited the content as needed and take(s) full responsibility for the content of the published article.

## Funding

This research was supported by the National Research Foundation of Korea (NRF) grant, funded by the Korea government (MSIT) (RS-2020-NR049559), a grant from the Korea Health Technology R&D Project through the Korea Health Industry Development Institute, funded by the Ministry of Health and Welfare (RS-2020-KH087790), the Basic Science Research Program through the National Research Foundation of Korea (NRF), funded by the Ministry of Education (RS-2020-NR054734), the Starting Growth Technological R&D Program (RS-2024-00507224) and a grant from the National Research Foundation of Korea, funded by the Korea government (RS-2024-00350362).

## Declaration of Competing Interest

The authors declare that they have no known competing financial interests or personal relationships that could have appeared to influence the work reported in this paper.

## Data Availability

No data was used for the research described in the article.
